# Host AKT-mediated phosphorylation of HIV-1 accessory protein Vif potentiates infectivity *via* enhanced degradation of the restriction factor APOBEC3G

**DOI:** 10.1016/j.jbc.2022.101805

**Published:** 2022-03-05

**Authors:** Rameez Raja, Chenyao Wang, Ritu Mishra, Arundhoti Das, Amjad Ali, Akhil C. Banerjea

**Affiliations:** 1 Department of Inflammation and Immunity, Cleveland Clinic, Cleveland, Ohio, USA; 2 Virology II, National Institute of Immunology, New Delhi, India; 3 Amity Institute of Virology and Immunology, Amity University, Noida, Uttar Pradesh, India; 4 Laboratory of Genome Integrity, National Cancer Institute, Bethesda, Maryland, USA; 5 Department of Molecular Cell and Cancer Biology, UMass Chan Medical School, Worcester, Massachusetts, USA

**Keywords:** HIV-1 Vif, phosphorylation, AKT, stabilization, APOBEC3G, AKTi, AKT inhibitor, APOBEC3G, apolipoprotein B mRNA-editing enzyme-catalytic polypeptide-like 3G, CBFβ, core binding factor β, cDNA, complementary DNA, CHX, cycloheximide, GST, glutathione-*S*-transferase, HA, hemagglutinin, HEK-293T, human embryonic kidney 293T cell line, His-Ub, histidine–Ub, KD-AKT, kinase-deficient AKT, LTR, long terminal repeat, MDM2, mouse double minute 2 homolog, NIH, National Institutes of Health, SAMHD1, SAM domain and HD domain–containing protein 1, Thr20, threonine 20, TRIM5, tripartite motif 5, Ub, ubiquitin, Vif, viral infectivity factor

## Abstract

HIV-1 encodes accessory proteins that neutralize antiviral restriction factors to ensure its successful replication. One accessory protein, the HIV-1 viral infectivity factor (Vif), is known to promote ubiquitination and proteasomal degradation of the antiviral restriction factor apolipoprotein B mRNA-editing enzyme-catalytic polypeptide-like 3G (APOBEC3G), a cytosine deaminase that leads to hypermutations in the viral DNA and subsequent aberrant viral replication. We have previously demonstrated that the HIV-1 viral transcription mediator Tat activates the host progrowth PI-3–AKT pathway, which in turn promotes HIV-1 replication. Because the HIV-1 Vif protein contains the putative AKT phosphorylation motif RMRINT, here we investigated whether AKT directly phosphorylates HIV-1 Vif to regulate its function. Coimmunoprecipitation experiments showed that AKT and Vif interact with each other, supporting this hypothesis. Using *in vitro* kinase assays, we further showed that AKT phosphorylates Vif at threonine 20, which promotes its stability, as Vif becomes destabilized after this residue is mutated to alanine. Moreover, expression of dominant-negative kinase-deficient AKT as well as treatment with a chemical inhibitor of AKT increased K48-ubiquitination and proteasomal degradation of HIV-1 Vif. In contrast, constitutively active AKT (Myr-AKT) reduced K48-ubiquitination of Vif to promote its stability. Finally, inhibition of AKT function restored APOBEC3G levels, which subsequently reduced HIV-1 infectivity. Thus, our results establish a novel mechanism of HIV-1 Vif stabilization through AKT-mediated phosphorylation at threonine 20, which reduces APOBEC3G levels and potentiates HIV-1 infectivity.

HIV-1 is a small retrovirus that manipulates the host proteins to survive and replicate inside the host by exploiting its cellular machinery (1, 2). HIV-1 genome contains nine ORFs, which encode 15 proteins; both structural and nonstructural genes. The nonstructural proteins include two regulatory proteins, Tat and Rev, and four accessory proteins, Vif, Vpr, Nef, and Vpu. HIV-1 accessory proteins play an important role in viral infectivity, gene expression, and pathogenicity by overcoming the host cell antiviral restriction factors. There are multiple anti-HIV-1 restriction factors like tripartite motif 5 (TRIM5) (3), apolipoprotein B mRNA-editing enzyme-catalytic polypeptide-like 3G (APOBEC3G) (4, 5), SAM domain and HD domain–containing protein 1 (SAMHD1) (6, 7), and tetherin (or bone marrow stromal antigen 2) (8). TRIM5 inhibits the uncoating of capsid proteins, which is required to release viral mRNA into host cell. SAMHD1 depletes the cellular pool of dNTPs to reduce HIV-1 replication. APOBEC3G is cytidine deaminase that converts cytosine to uracil. These mutations lead to the degradation of proviral complementary DNA (cDNA), and even if the cDNA is integrated into host genome, it is rendered nonfunctional because of the mutations. Tetherin binds to the surface of cell at the site of budding virions and blocks the release of viral progeny. HIV-1 has developed various strategies to evade these restriction factors except TRIM5 as none of the HIV-1 proteins is known to target it. The restriction factors SAMHD1, tetherin, and APOBEC3G are targeted by Vpx/Vpr, Vpu (or Env), and Vif, respectively (9).

HIV-1 Vif plays an important role in viral survival in nonpermissive APOBEC3G-expressing host cells including primary T-lymphocytes and macrophages. To counter the APOBEC3G-mediated antiviral effect in these cells, Vif recruits E3 ubiquitin (Ub) ligase complex consisting CUL5, ELOB/C, RBX, and core binding factor β (CBFβ) for APOBEC3G degradation *via* proteasomal pathway (10, 11, 12, 13). Vif-deficient viruses are severely compromised and unable to multiply in host cells. The cellular proteins regulating the Vif activity have profound effect on HIV-1 pathogenesis. Mouse double minute 2 (MDM2) homolog, an E3 ligase, has been shown to interact with Vif leading to its ubiquitination followed by its proteasomal degradation, which results in an increase of APOBEC3G level (14). CBFβ on the other hand is known to stabilize Vif and hence counteract antiviral effect of APOBEC3G (10, 15). However, apoptosis signal–regulating kinase-1 disrupts the interaction between Vif and APOBEC3G to restore the antiviral activity of APOBEC3G (16).

HIV-1 Tat protein is already known to play an important role in the activation of PI3K–AKT signaling pathway (17, 18). MDM2 is a downstream target of AKT (19), and we have previously shown that HIV-1 Tat protein stabilizes MDM2 by inducing its phosphorylation in AKT-dependent manner (18). In addition, MDM2 is known to enhance the Tat-mediated long terminal repeat (LTR) activity by ubiquitinating Tat at lysine 71 position to potentiate its activity in a nonproteolytic way (20). Thus, there is a positive feedback loop between Tat, AKT, and MDM2. Since MDM2 ubiquitinates HIV-1 Vif protein to induce its proteasomal degradation and lies downstream in the AKT signaling pathway, we investigated the role of AKT in regulating the HIV-1 Vif levels. Furthermore, the residues surrounding threonine 20 (Thr20) of HIV-1 Vif (RMRINT) resemble the AKT phosphorylation site (RXRXXS/T); similar motifs have been found in AKT target substrates like FKRHL1 (a member of the Forkhead transcription factor family), IκB kinase α, and P21 (19, 21). AKT-mediated phosphorylation in these substrate proteins regulates their function. We report here that AKT stabilizes Vif protein level to promote APOBEC3G degradation and enhances HIV-1 infectivity. This study can have significant implications toward a better understanding of HIV-1 pathogenesis.

## Results

### Screening of viral genes as a target substrate for AKT

**Figure 1:**
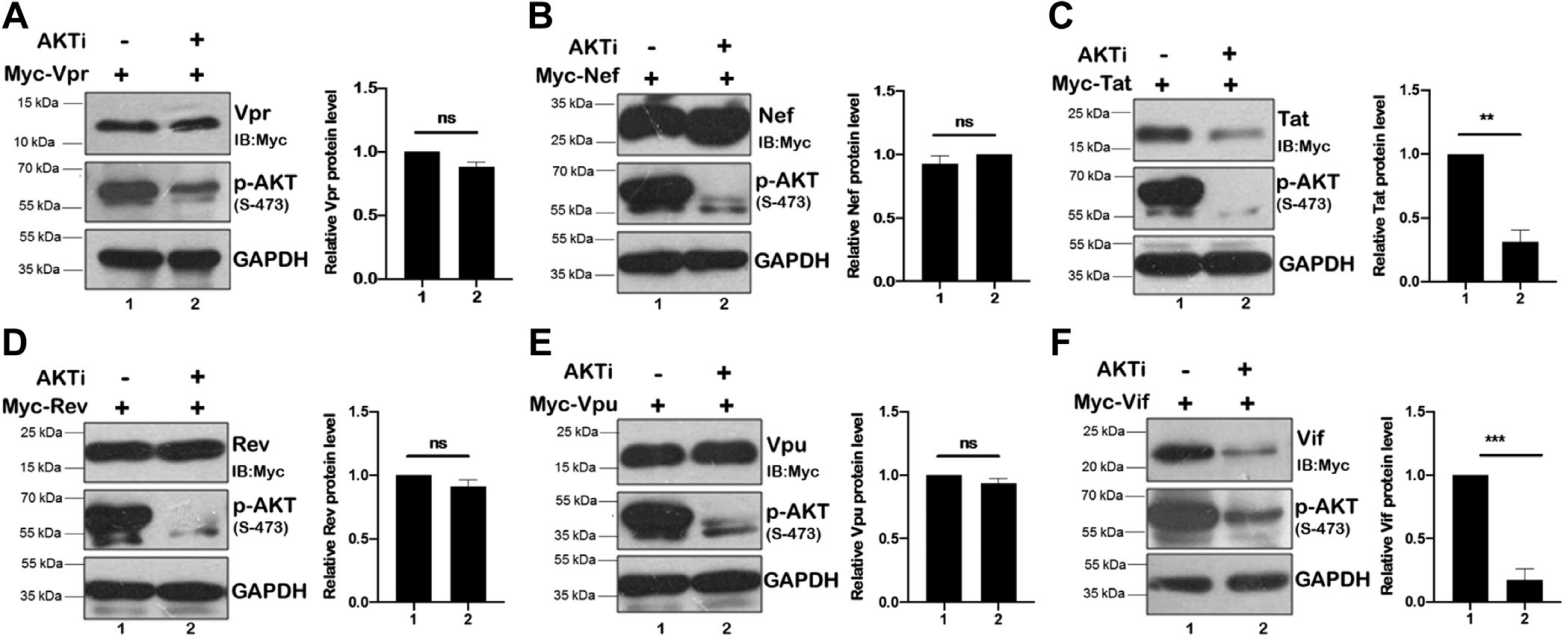
**Screening of HIV-1 genes.***A*–*F*, HEK-293T cells were transfected with Myc-Vpr, Myc-Nef, Myc-Tat, Myc-Rev, Myc-Vpu, and Myc-Vif plasmids for 36 h. AKT inhibitor treatment was given for 8 h before harvesting cells. Cell lysates were analyzed by Western blotting using anti-Myc, anti-p-AKT, and anti-GAPDH antibodies. GAPDH was used as loading control. *Bar plots* represent the quantification and statistical analysis of Western blots (mean ± SEM, n = 3; ns > 0.05; ∗*p* < 0.05; ∗∗*p* < 0.01, and ∗∗∗*p* < 0.001). HEK-293T, human embryonic kidney 293T cell line; ns, not significant.

To investigate the effect of AKT on expression of HIV-1 accessory genes and regulatory genes, Myc-tagged viral genes including Tat, Rev, Nef, Vpu, Vpr, and Vif were expressed in human embryonic kidney 293T (HEK-293T) cells followed by the treatment with a chemical inhibitor of AKT (AKTi). The expression level of these viral proteins was monitored by immunoblotting using anti-Myc antibody (Fig. 1, *A*–*D* and *F*). From our screening experiments, we identified that the expression of two viral proteins namely, HIV-1 Tat and HIV-1 Vif, was reduced in the presence of AKT inhibitor (Fig. 1, *C* and *F*), whereas the expression of Vpr, Nef, Rev, and Vpu remained unaffected (Fig. 1, *A*, *B*, *D* and *E*). These data suggest that AKT regulates Tat and Vif, which can have significant consequences on Tat-AKT-Mdm2 axis and hence HIV-1 pathogenesis.

### AKT inhibition decreases HIV-1 Vif protein level in a dose-dependent manner

**Figure 2:**
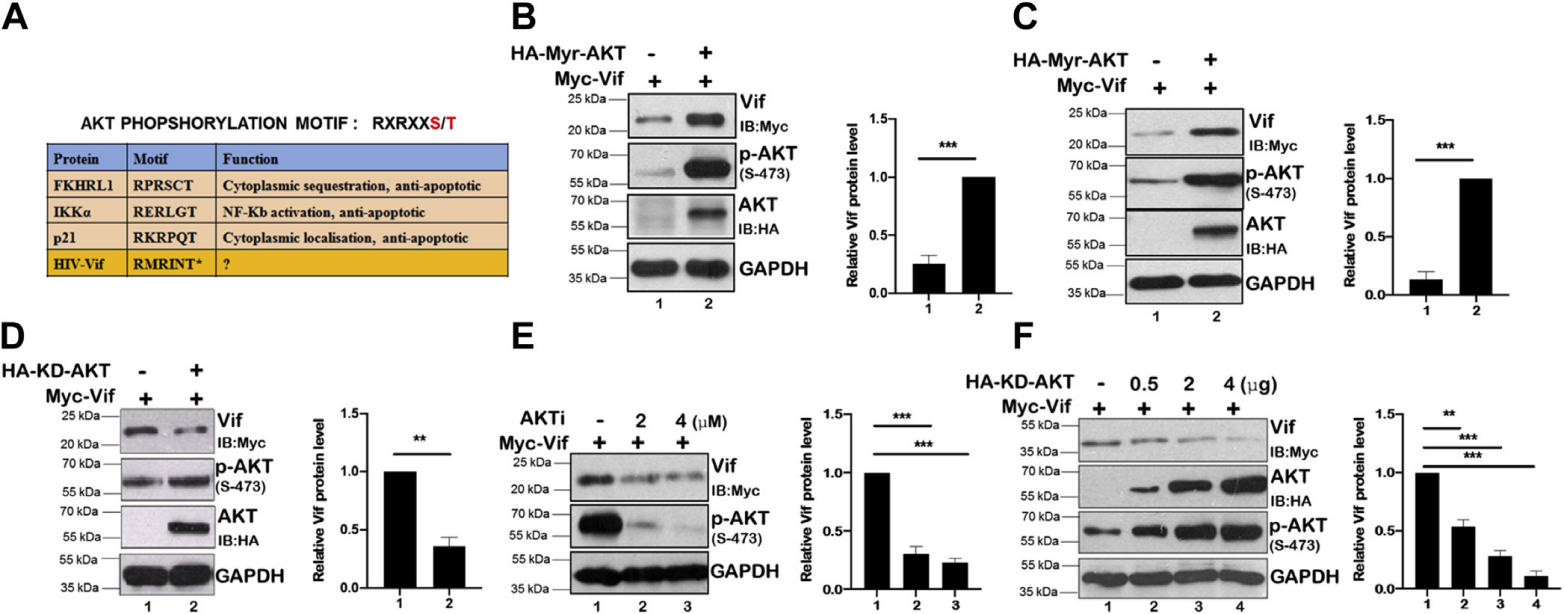
**AKT inhibition decreases HIV-1 Vif protein level in a dose-dependent manner.***A*, the table showing AKT phosphorylation motif in different proteins and HIV-1 Vif protein. *B*, HEK-293T cells were cotransfected with Myc-Vif and HA-Myr-AKT as indicated. After 24 h of transfection, cells were lysed and cell lysates were subjected to Western blotting using anti-HA, anti-phospho-AKT Ser-473, anti-Myc, and anti-GAPDH antibodies. GAPDH was used as loading control. *C*, MCF-7 cells were cotransfected with Myc-Vif and HA-Myr-AKT as indicated. After 24 h of transfection, cells were lysed and cell lysates were subjected to Western blotting using anti-HA, anti-phospho-AKT Ser-473, anti-Myc, and anti-GAPDH antibodies. GAPDH was used as loading control. *D*, HEK-293T cells were cotransfected with Myc-Vif and HA-KD-AKT as indicated. After 24 h of transfection, cells were lysed and cell lysates were subjected to Western blotting using anti-HA, anti-phospho-AKT Ser-473, anti-Myc, and anti-GAPDH antibodies. GAPDH was used as loading control. *E*, HEK-293T cells were transfected with Myc-Vif for 24 h followed by AKTi treatment of 2 and 4 μM for 8 h. Cells were lysed, and cell lysates were subjected to Western blotting using anti-HA, anti-phospho-AKT Ser-473, anti-Myc, and anti-GAPDH antibodies. GAPDH was used as loading control. *F*, HEK-293T cells were transfected with Myc-Vif along with different concentrations of HA-KD-AKT for 24 h. Cells were lysed, and cell lysates were subjected to Western blotting using anti-HA, anti-phospho-AKT Ser-473, anti-HA, anti-Myc, and anti-GAPDH antibodies. GAPDH was used as loading control. *Bar plots* represent the quantification and statistical analysis of Western blots (mean ± SEM, n = 3; ns > 0.05; ∗*p* < 0.05; ∗∗*p* < 0.01, and ∗∗∗*p* < 0.001). AKTi, AKT inhibitor; HA, hemagglutinin; HEK-293T, human embryonic kidney 293T cell line; KD-AKT, kinase-deficient AKT; ns, not significant.

Because HIV-1 Vif has a putative AKT phosphorylation motif RMRINT and its protein level was reduced by AKTi treatment (Figs. 2*A* and 1*F*), here we studied its regulation in more detail. However, the consequences of AKT-mediated Tat regulation remain to be explored. To test the effect of AKT on Vif protein level, HEK-293T and MCF-7 were transfected either with Myc-Vif alone or along with constitutively active form of AKT (Myr-AKT) followed by analysis of Vif protein level by immunoblotting. A significant increase in Vif levels was observed in the presence of Myr-AKT in both these cell lines (Fig. 2, *B* and *C*). In contrast, the level of Vif was significantly reduced in the presence of kinase-deficient AKT (KD-AKT), indicating that AKT activity controls Vif protein level (Fig. 2*D*). HIV-1 Vif protein level was also investigated in the presence of different concentrations of AKTi at 2 and 4 μM. Both the doses reduced the HIV-Vif protein level, but 4 μM dose showed the maximum effect (Fig. 2*E*). Similarly, dose-dependent inhibition of AKT activity on HIV-1 Vif was observed more prominently by using KD-AKT. Myc-Vif was cotransfected along with different concentrations of KD-AKT in HEK-293T cells. KD-AKT was able to decrease the HIV-1 Vif protein level in a dose-dependent manner (Fig. 2*F*). Thus, these data indicate that AKT regulates the level of Vif protein.

### AKT regulates Vif protein at post-translational level

**Figure 3:**
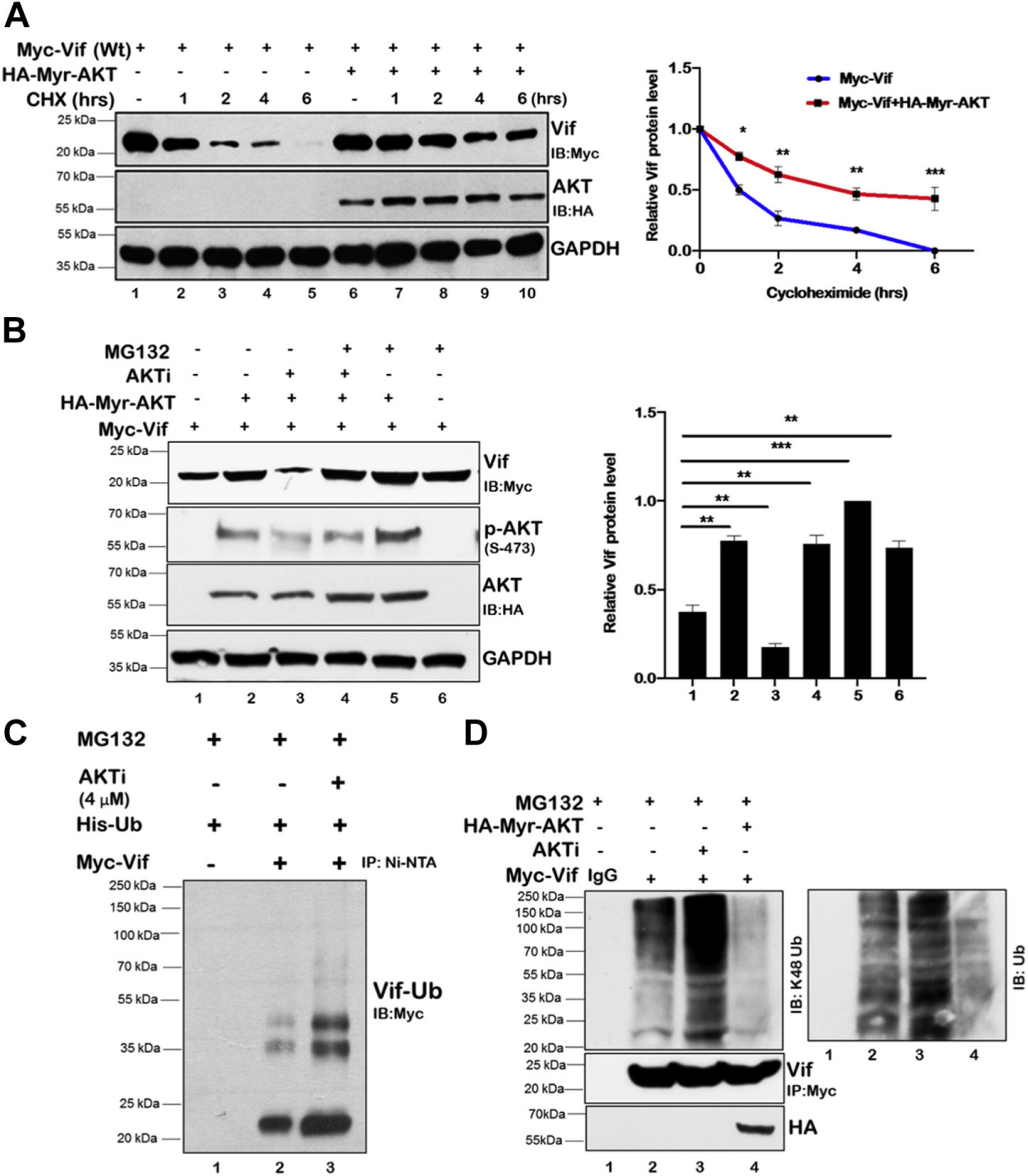
**AKT stabilizes Vif expression at post-translational level.***A*, HEK-293T cells were transfected with either Myc-Vif alone or along with HA-Myr-AKT for 36 h. Cells were treated with cycloheximide (100 μg/ml) for the indicated periods. Cell lysates were subjected to Western blot analysis using anti-HA, anti-Myc, and anti-GAPDH antibodies. GAPDH was used as loading control. Densitometric analysis was done by ImageJ and shown as *line graph*. *B*, HEK-293T cells were cotransfected with HA-Myr-AKT and Myc-Vif plasmids for 24 h and then treated with AKTi (4 μM). After 24 h, cells were treated with MG132 (10 μM) for 8 h. Cell lysates were analyzed by Western blotting using anti-HA, anti-Myc, anti-phospho-AKT Ser-473, and anti-GAPDH antibodies. GAPDH was used as loading control. *C*, HEK-293T cells were transfected with Myc-Vif and His-Ub expression plasmids and treated with AKTi (4 μM). After 24 h, cells were treated with MG132 (10 μM) for 8 h. Cell lysates were subjected to immunoprecipitation with Ni–NTA beads followed by Western blotting using anti-Myc antibody. *D*, HEK-293T cells were transfected with Myc-Vif and Myr-AKT expression plasmids for 24 h and then treated with AKTi (4 μM). Cells were treated with MG132 (10 μM) for 8 h. Cells were lysed and subjected to immunoprecipitation assay with Myc antibody and analyzed by Western blotting using K48 and total Ub antibodies. *Bar plots* represent the quantification and statistical analysis of Western blots (mean ± SEM, n = 3; ns > 0.05; ∗*p* < 0.05; ∗∗*p* < 0.01, and ∗∗∗*p* < 0.001). AKTi, AKT inhibitor; HA, hemagglutinin; HEK-293T, human embryonic kidney 293T cell line; Ni–NTA, nickel–nitrilotriacetic acid; ns, not significant; Ub, ubiquitin.

After confirming that HIV-1 Vif protein is a target for AKT, we inquired whether the effect is at transcriptional or post-translational level by using cycloheximide (CHX). CHX inhibits new protein synthesis and hence is used to assess the stability of protein at post-translational level. Myc-Vif was transfected either alone or along with hemagglutinin (HA)-Myr-AKT in HEK-293T cells. In the presence of Myr-AKT, Vif protein levels were observed to be more stable even after longer time duration of CHX treatment (Fig. 3*A*). These results suggest that Vif protein is regulated by AKT at post-translational level.

In order to assess the involvement of proteasomal pathway in Vif protein regulation by AKT, HEK-293T cells were transfected with either Myc-Vif alone or along with Myr-AKT followed by treatment with AKTi or MG132 (proteasomal inhibitor). The cells were harvested and subjected to immunoblotting to analyze the Vif protein level. As expected, Vif protein level increased in the presence of the Myr-AKT and decreased in AKTi-treated cells. Interestingly, Vif protein degradation was rescued in MG132-treated cells, indicating the role of proteasomal degradation pathway in the process (Fig. 3*B*).

As proteasomal pathway was found to play a role in AKT-mediated regulation of Vif protein levels, we also assessed the ubiquitination levels of Vif protein in the presence of AKTi with MG132 treatment in all samples. MG132 blocks proteasome pathway and prevents K48-ubiquitinated proteins from degradation. HEK-293T cells were transfected with Myc-Vif and histidine–Ub (His-Ub) followed by treatment with AKTi. These cells were then further treated with MG132, and total ubiquitinated proteins were detected by immunoblotting after pull down with nickel–nitrilotriacetic acid beads. As expected, Vif ubiquitinylation increased in the presence of AKTi indicating a role of AKT in post-translational regulation of Vif (Fig. 3*C*). To further validate the role of AKT in regulating Vif protein level through proteasome pathway, we also analyzed Vif protein K-48 ubiquitination either in the presence of AKTi or in the presence of Myr-AKT. We observed that inhibition of AKT activity by AKTi treatment increased K-48 ubiquitination profile of Vif, whereas Myr-AKT reduced Vif K-48 ubiquitination (Fig. 3*D*). These data clearly indicate that AKT regulates the stability of HIV-1 Vif protein by decreasing its K-48 ubiquitination and hence proteasomal degradation.

### HIV-1 Vif interacts with AKT and is phosphorylated by it

**Figure 4:**
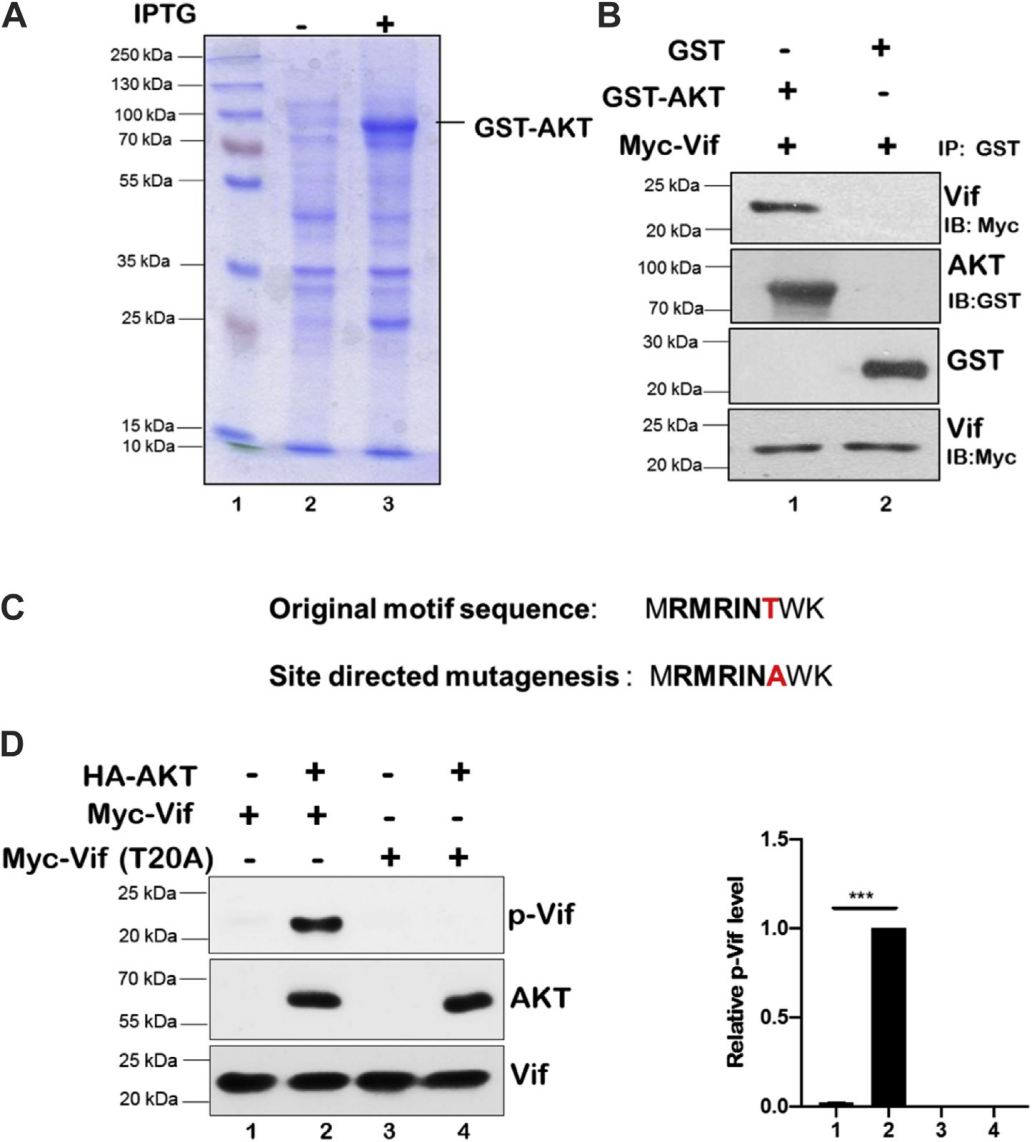
**HIV-1 Vif interacts with AKT and is phosphorylated by it.***A*, GST-AKT was purified from bacterial system using IPTG induction as described in the Experimental procedures section. *B*, HEK-293T cells were transfected with Myc-Vif encoding plasmid. After 24 h of transfection, cell lysates were prepared and incubated with GST-AKT fusion protein and GST alone bound to GST beads for 24 h at 4 °C. The bound proteins were analyzed by Western analysis using Myc antibody. GST alone was used as control. *C*, HIV-1 Vif has RMRINT motif (AKT phosphorylation motif). The RMRINA point mutant Myc-Vif (T20A) was generated using site-directed mutagenesis. *D*, HEK-293T cells were transfected with HA-AKT, Myc-Vif, and mutant Myc-Vif (T20A). AKT and Vif proteins were purified by immunoprecipitation. The purified AKT was added to the purified Vif and its mutant (T20A) along with kinase reaction buffer. The phosphorylated AKT substrates were analyzed by Western blotting. *Bar plots* represent the quantification and statistical analysis of Western blots (mean ± SEM, n = 3; ns > 0.05; ∗*p* < 0.05; ∗∗*p* < 0.01, and ∗∗∗*p* < 0.001). GST, glutathione-*S*-transferase; HEK-293T, human embryonic kidney 293T cell line; ns, not significant.

After establishing that HIV-1 Vif is regulated by AKT, we determined whether the two proteins interact with each other. To investigate the interaction of HIV-1 Vif with AKT, glutathione-*S*-transferase (GST)-AKT was purified from the bacterial expression system using BL21 cells as shown in Figure 4*A*. Purified GST-AKT or GST bound to glutathione beads was used to pull down the Vif protein from Myc-Vif-transfected cell lysates. GST-AKT, but not GST, showed a strong interaction with Vif protein as shown in Figure 4*B*. AKT, being a kinase, is known to phosphorylate its target proteins having consensus AKT phosphorylation motif RXRXXS/T. HIV-1 Vif has an AKT phosphorylation motif RMRINT in its sequence, which is similar to the consensus RXRXXS/T motif. Therefore, we performed kinase assay to determine the role of this motif in Vif protein phosphorylation. Thr20 residue present in the HIV-1 Vif phosphorylation motif RMRINT was mutated to alanine (T20A) from Myc-Vif Wt construct by site-directed mutagenesis (Fig. 4*C*) and was used as a control to validate the role of Thr residue in Vif phosphorylation. Indeed, we found that HIV-1 Vif Wt was phosphorylated by AKT, whereas HIV-1 Vif T20A mutant was not (Fig. 4*D*). These data indicate that HIV-1 Vif is phosphorylated by AKT at T20 position.

### Vif mutant T20A is less stable than Vif Wt

**Figure 5:**
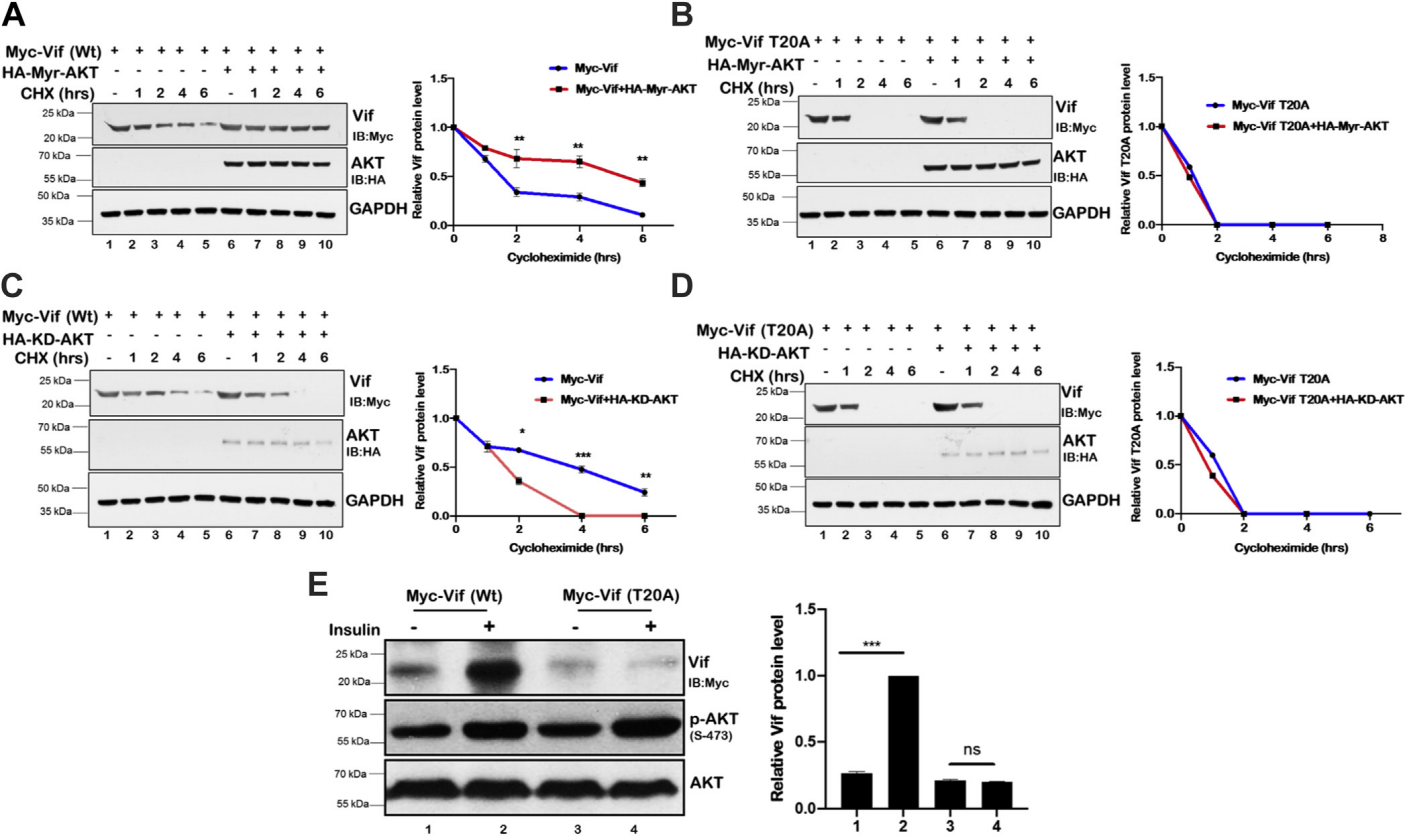
**Vif mutant T20A is less stable than Vif Wt.***A* and *B*, HEK-293T cells were transfected with Myc-Vif or Myc-Vif (T20A) expression plasmids either alone or in the presence of Myr-AKT for 36 h and then treated with CHX (100 μg/ml) for indicated periods. Cell lysates were analyzed by Western blotting with anti-Myc and anti-GAPDH antibodies. GAPDH was used as loading control. Densitometric analysis was done by ImageJ and shown as *line graph* control. *C* and *D*, HEK-293T cells were transfected with Myc-Vif and Myc-Vif (T20A) expression plasmids either alone or in the presence of KD-AKT as indicated for 36 h. Cell lysates were analyzed by Western blotting with anti-Myc and anti-GAPDH antibodies. GAPDH was used as loading control. Densitometric analysis was done by ImageJ and shown as *line graph* control. *E*, HEK-293T cells were transfected with Myc-Vif and Myc-Vif (T20A) expression plasmids for 36 h and then treated with insulin. Cell lysates were subjected to Western blotting analysis using anti-Myc, anti-phospho-AKT Ser-473, and anti-AKT antibodies. AKT was used as internal loading. *Bar plots* represent the quantification and statistical analysis of Western blots (mean ± SEM, n = 3; ns > 0.05; ∗*p* < 0.05, ∗∗*p* < 0.01, and ∗∗∗*p* < 0.001). CHX, cycloheximide; HEK-293T, human embryonic kidney 293T cell line; KD-AKT, kinase-deficient AKT; ns, not significant.

From our previous results, we identified that Thr20 of Vif is the target site for AKT-mediated phosphorylation. To study the role of T20 phosphorylation in AKT-mediated Vif protein stability, Myc-Vif Wt and Myc-Vif T20A were expressed in HEK-293T cells either alone or along with Myr-AKT or KD-AKT followed by CHX-chased assay for different time points. The protein levels of Vif Wt and Vif T20A were measured by immunoblotting using anti-Myc antibody. The mutant Vif (T20A) showed faster degradation kinetics compared with Wt (Fig. 5, *A* and *B*). Moreover, Myr-AKT stabilized Myc-Vif Wt, whereas KD-AKT promoted its degradation (Fig. 5, *A* and *C*), whereas, Myr-AKT or KD-AKT showed no effect on Myc-Vif T20A (Fig. 5, *B* and *D*) suggesting that Thr20 of HIV-1 Vif is important for AKT-mediated Vif protein stabilization (Fig. 5, *A*–*D*). These results further confirm the role of AKT in mediating Vif stabilization.

### Insulin increases protein level of Vif Wt but not Vif (T20A) mutant

Insulin is a growth factor that stimulates PI3K–AKT signaling pathway. We therefore inquired if insulin treatment can modulate Vif protein levels and thus validate our previous results. HEK-293T cells were transfected with either Myc-Vif or Myc-Vif (T20A) mutant to express these proteins and then treated with insulin. Vif protein levels were measured by immunoblotting. HIV-1 Vif Wt protein level was found to be increased in the presence of insulin, whereas the protein level of Vif (T20A) mutant remained unaffected by insulin treatment (Fig. 5*E*). These data further confirm our finding that AKT stabilizes Vif, which is dependent on its Thr20 residue.

### Downregulation of Vif by AKT inhibition restores APOBEC3G levels

**Figure 6:**
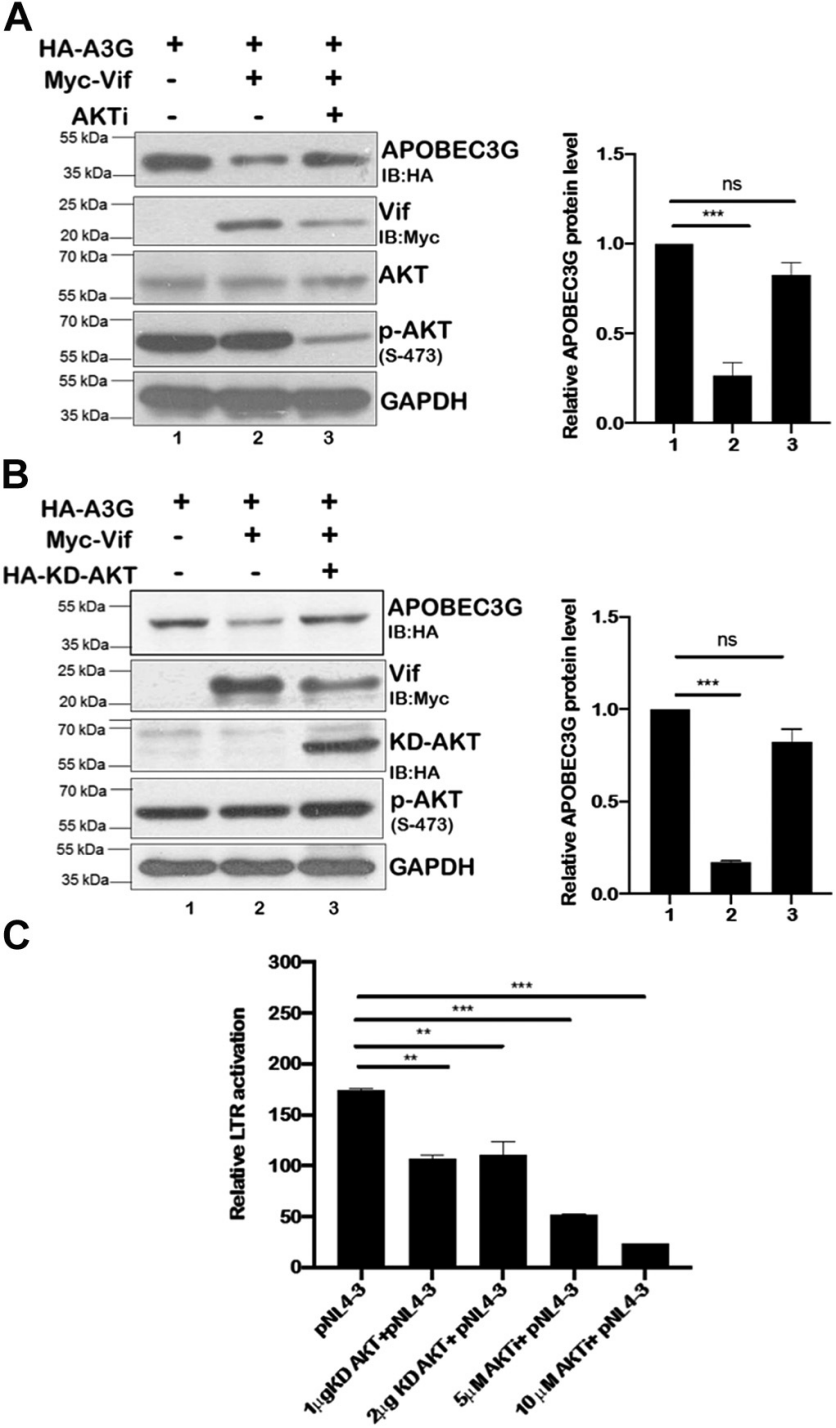
**Downregulation of Vif by AKT inhibition restores APOBEC3G levels.***A*, HEK-293T cells were cotransfected with HA-APOBEC3G and Myc-Vif as indicated and then treated with AKTi (4 μM) after 36 h of transfection. Cell lysates were subjected to SDS-PAGE followed by Western blotting using anti-HA, anti-Myc, anti-phospho-AKT Ser-473, anti-AKT, and anti-GAPDH antibodies. GAPDH was used as loading control. *B*, HEK-293T cells were cotransfected with HA-APOBEC3G, Myc-Vif, and HA-KD-AKT as indicated for 36 h. Cells were lysed, and cell lysates were analyzed by SDS-PAGE followed by Western blotting using anti-HA, anti-Myc, anti-phospho-AKT Ser-473, and anti-GAPDH antibodies. GAPDH was used as loading control. *C*, HEK-293T cells expressing APOBEC3G were either cotransfected with KD-AKT and pNL4-3 or pNL4-3 alone and then treated with AKTi. Treatment with AKTi continued for 72 h post-transfection. HEK-293T culture supernatant containing HIV-1 virions was used to infect TZMbl cells and measure HIV-1 infectivity by determining luciferase activity. *Bar plots* represent the quantification and statistical analysis of Western blots (mean ± SEM, n = 3; ns > 0.05; ∗*p* < 0.05, ∗∗*p* < 0.01, and ∗∗∗*p* < 0.001). AKTi, AKT inhibitor; APOBEC3G, apolipoprotein B mRNA-editing enzyme-catalytic polypeptide-like 3G; HA, hemagglutinin; HEK-293T, human embryonic kidney 293T cell line; KD-AKT, kinase-deficient AKT; ns, not significant.

The functional consequences of the AKT-mediated stabilization of Vif were investigated on APOBEC3G and HIV-1 infectivity. HIV-1 Vif protein is known to induce proteasomal degradation of APOBEC3G and enhance HIV-1 infectivity. To demonstrate this, we expressed HA-APOBEC3G alone or along with Myc-Vif in HEK-293T cells followed by AKTi treatment. Myc-Vif and APOBEC3G levels were monitored by immunoblotting. Expression of Myc-Vif reduced HA-APOBEC3G level significantly. However, the cells treated with AKTi showed reversion in APOBEC3G levels even in the presence of Vif. This reversion was seen because of AKTi-mediated downregulation of Vif (Fig. 6*A*). These results were also validated with KD-AKT. HEK-293T cells were transfected with either HA-APOBEC3G alone or along with Myc-Vif and HA-KD-AKT followed by immunoblotting to measure Vif and APOBEC3G protein levels. KD-AKT was found to restore the APOBEC3G level by downregulating Vif protein level (Fig. 6*B*). As our results demonstrated the role of AKT in regulating Vif protein level and hence APOBEC3G, we next investigated the effect of AKT on HIV-1 infectivity using TZMbl cells. These cells have luciferase downstream HIV-1 LTR HIV-1, and hence, luciferase expression can be used to measure HIV-1 infectivity. HIV-1 viral supernatant was collected from HEK-293T cells either in the presence of KD-AKT or AKTi and then used to infect TZMbl cells. We observed that the inhibition of AKT activity by KD-AKT or AKTi resulted in decrease of HIV-1 infectivity when the virus was produced in the presence of APOBEC3G (Fig. 6*C*), thereby supporting our earlier results.

## Discussion

HIV-1 has evolved various mechanisms to counter host cell antiviral restriction factors like TRIM5 (3), APOBEC3G (4, 5), SAMHD1 (6, 7) and tetherin (or bone marrow stromal antigen 2) (8). APOBEC3G induces hypermutations in viral DNA by cytidine deaminase activity leading to degradation of viral DNA. However, if hypermutated viral DNA gets integrated in the cellular genome, it cannot code for functional viral proteins (9). HIV-1 relies on Vif protein to overcome the potent antiviral function of APOBEC3G. HIV-1 Vif recruits an E3-Ub ligase complex to induce proteasomal pathway–dependent degradation of APOBEC3G and prevents its incorporation in the newly formed virions.

However, there are very few reports of cellular proteins known to regulate HIV-1 Vif protein, for example, CBFβ is known to stabilize Vif and counter the APOBEC3G levels (15) while apoptosis signal–regulating kinase-1 helps to restore APOBEC3G level by disrupting Vif-APOBEC3G interaction (16). Recently, we demonstrated that Vif is degraded by carboxyl-terminus of Hsp70-interacting protein (22). On the other hand, MDM2 is reported to promote Vif degradation and elevate APOBEC3G levels (14). MDM2 is a downstream target of AKT, and we have previously shown that in HIV-infected cells, AKT enhances HIV-1 replication through Tat-AKT-MDM2 loop by stabilizing MDM2 (18). Because HIV-1 Vif has a putative AKT phosphorylation motif (RMRINT) and AKTi treatment reduced Vif protein level, we investigated the role of AKT in regulation of HIV-1 Vif. Inhibition of AKT activity by using AKTi or dominant negative form of AKT (KD-AKT) was found to reduce Vif protein level. In contrast, expression of the constitutively active form of AKT (Myr-AKT) significantly increased the Vif protein level, which was also confirmed by CHX-chase assay. Level of Vif protein reduction in response to AKTi was found to be proteasome pathway dependent, as proteasome inhibition by MG132 restored Vif protein level. *In vivo* ubiquitination assay further showed that increase of AKT activity by Myr-AKT reduces total as well as K48-linked Vif ubiquitination, whereas inhibition of AKT function by AKTi increases the total and K48-linked ubiquitination of Vif. Thus, the ubiquitination assay clearly demonstrated that AKT activity prevents Vif from proteasomal degradation by inhibiting its K48 ubiquitination. The functional effect of AKT-mediated regulation of Vif protein was also observed on Vif-induced degradation of APOBEC3G, which was reverted by inhibition of AKT function through AKTi or KD-AKT. These results suggest that AKT increases the level of HIV-1 Vif protein and helps the virus in combating APOBEC3G. Furthermore, HIV infectivity was reduced in the presence of KD-AKT and AKTi indicating the role of AKT in modulating Vif and APOBEC3G levels. However, the ectopic concentration of different proteins used in our cell culture studies does not represent the physiological concentration. It would be an interesting aspect to study the role of AKT in regulating Vif and APOBEC3G proteins and hence HIV-1 pathogenesis in HIV-1-infected patient samples.

Mechanistically, AKT was found to interact with Vif as shown by GST pull-down assay. Moreover, Thr20 residue present in AKT phosphorylation motif of Vif was found to be important for AKT-mediated Vif phosphorylation as well as its stabilization. Vif T20A protein levels remained unaffected by either activation of AKT activity by Myr-AKT or its inhibition by KD-AKT or AKTi. Similarly, activation of AKT pathway by insulin treatment led to the increase in Vif protein level but failed to increase Vif-T20A protein level, thereby confirming the central role of Thr20 in regulation of Vif protein by AKT pathway.

**Figure 7:**
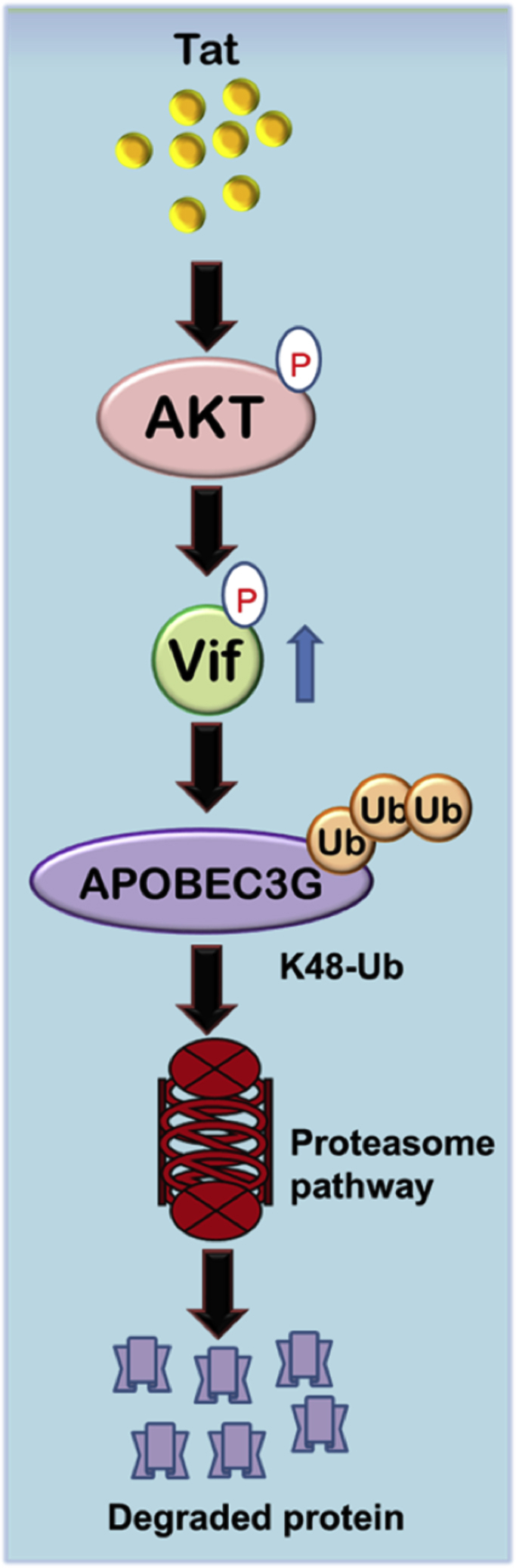
**Mechanistic model showing Tat-AKT-Vif axis to regulate APOBEC3G levels.** HIV-1 Tat activates AKT, and activated AKT in turn phosphorylates Vif to stabilize it, which then targets APOBEC3G for degradation to promote viral replication. APOBEC3G, apolipoprotein B mRNA-editing enzyme-catalytic polypeptide-like 3G.

The exploitation and modulation of host cellular signaling pathways by HIV-1 proteins is a very complex phenomenon. HIV-1 Tat is an early expressed protein of the virus, which activates AKT signaling pathway (17, 18). Activated AKT phosphorylates MDM2 to stabilize it as previously shown by us resulting in K63-linked Tat ubiquitination to increase the LTR transcriptional activity, thus creating a positive feedback loop between Tat, AKT, and MDM2 (18, 20). Vif is ubiquitinated and degraded by MDM2, which is activated by AKT itself. However, our results presented here show that AKT also stabilizes Vif protein through its phosphorylation at Thr20 residue. Thus, HIV-1 exploits AKT signaling pathway through its activation by Tat resulting in the enhancement of Vif protein levels. It helps the virus to evade the host restriction factor APOBEC3G more effectively (Fig. 7) and possibly to overcome the degradation of Vif by MDM2. This study can have significant implications as HIV-1 Tat protein and growth factors like insulin activate PI3K–AKT kinase pathway and can potentially modulate Vif and APOBEC3G protein levels and hence HIV-1 pathogenesis.

## Experimental procedures

### Cell culture and transfection

HEK-293T, MCF-7, and TZMbl were maintained in Dulbecco’s modified Eagle’s medium (Himedia Laboratories) supplemented with 10% fetal bovine serum (Gibco, Invitrogen), 100 units of penicillin, 0.1 mg of streptomycin, and 0.25 μg of amphotericin B per ml at 37 °C in the presence of 5% CO_2_ in a humidified incubator. Transfections were performed using Lipofectamine 2000 (Invitrogen) and linear polyethyleneimine (molecular weight of 25,000; Polysciences, Inc) reagents using the manufacturer’s protocol.

### Plasmid constructs and chemicals

Viral genes were obtained from the National Institutes of Health (NIH)-AIDS reagent inventory as previously described (23). HA-APOBEC3G was cloned using cDNA from TZMbl cells in pCMV-HA vector from Clontech. pBlue3′LTR-luc was obtained from NIH AIDS Reference and Reagent Program of NIH. Myc-Vif (T20A) was made from Wt construct by site-directed mutagenesis. HA-AKT, HA-KD-AKT (K179M), HA-Myr-AKT, and GST-AKT were a kind gift from Hui Kuan Lin, MD Anderson Cancer Centre. Renilla luciferase plasmid was a kind gift from Vivek Natarajan, Institute of Genomics and Integrative Biology. His-Ub plasmid was gifted by Dimitris Xirodimas, University of Dundee. Chemicals used were AKTi (Sigma), IPTG (Sigma), insulin (Sigma), CHX (Sigma), and MG132 (Sigma).

### Western analysis

HEK-293T cells were transfected with the gene of interest for either 24 h or 36 h. The cells were harvested and lysed in radioimmunoprecipitation assay lysis buffer (1% NP-40, 20 mM Tris-Cl, pH 7.5, 150 mM NaCl, 1 mM Na_2_EDTA, 1 mM EGTA, 1% sodium deoxycholate, and 1 mM Na_3_VO_4_). Protein estimation was carried out by using BCA Protein Assay Kit (Pierce, Thermo Scientific). An equal amount of protein was loaded on SDS-PAGE and transferred to nitrocellulose membrane as described before (24). The membranes were blocked with 5% nonfat dry milk (Himedia Laboratories) or 5% bovine serum albumin. The primary antibodies used were anti-AKT, anti-GAPDH, anti-phospho-AKT (S-473) (Cell Signaling Technology), anti-Myc, anti-HA (Clontech), ab140601 (abcam–K48 antibody), and anti-GST (Santa Cruz Biotechnology). The secondary antibodies used were anti-rabbit/mouse-horseradish peroxidase conjugated (Jackson ImmunoResearch). Blots were developed using Enhanced Chemiluminescence) reagent.

### CHX-chase assay

To study the degradation kinetics of proteins, CHX-chase assay was performed. HEK-293T cells were transfected with the gene of interest for 24 h and then treated with CHX (100 μg/ml; Sigma). Cell lysates were prepared at indicated time points and subjected to 10% SDS-PAGE followed by Western analysis as described previously.

### *In vivo* ubiquitination assay

*In vivo* ubiquitination assay was performed to detect ubiquitylated proteins in transfected mammalian cells as described earlier (25, 26, 27). HEK-293T cells were cotransfected with plasmid encoding the desired gene and 6&times His-Ub for 36 h. After 36 h of transfection, 10 μM of MG132 (Sigma–Aldrich) was added, and the cells were further incubated for 8 h. The cells were lysed in buffer A (6 M guanidinium HCl, 0.1 M Na_2_HPO_4_/NaH_2_PO_4_, 10 mM imidazole; pH 8.0), sonicated, and centrifuged. Nickel–nitrilotriacetic acid beads were added to the supernatant, and the mixture was incubated at room temperature for 6 h while rotating. Subsequently, the beads were washed with buffer A and 25 mM Tris, pH 6.8, and 20 mM imidazole buffer. The ubiquitinated proteins were eluted in buffer containing 200 mM imidazole, 5% SDS, 0.15 M Tris, pH 6.7, 30% glycerol, and 0.72 M β-mercaptoethanol. The eluates were resolved by SDS-PAGE followed by Western blot analysis.

### Site-directed mutagenesis and sequencing

To generate Myc-Vif with mutation in RMRINT motif, site-directed mutagenesis was carried out by using Quik Change II Site-Directed Mutagenesis Kit (Agilent). The primers used were:

5′-GTAGACAGGATGAGGATTAACGCCTGGAAAAGATTAGTAAAACAC-3’ (forward primer) and 5′-GTGTTTTACTAATCTTTTCCAGGCGTTAATCCTCATCCTGTCTAC-3’ (reverse primer). The PCR conditions were 95 °C (1 min), 95 °C (50 s), 60 °C (50 s), 95 °C (5 min), and 95 °C (7 min). After thermal cycling, DpnI treatment was given to digest parental and hemimethylated DNA followed by the transformation in competent cells. The sequencing was done by SciGenome Labs, India. The clone positive for the mutation as shown by sequencing results was used for the experiments.

### GST-AKT expression and purification

pGEX-4T1-AKT was transformed in BL21 strain of *Escherichia coli* for expression and subsequent purification of GST-AKT. The bacterial culture was induced with 0.5 mM IPTG for 16 h at 16 ^°^C. The cells were lysed by adding lysozyme (1 mg/ml) at 4 ^°^C with gentle shaking. DTT was added to the bacterial lysate after lysozyme treatment (100 μl of 1 M DTT). This was followed by sonication and extraction of proteins with Triton X-100. The solution was centrifuged at 12,000 rpm for 15 min at 4 ^°^C. The supernatant was then used for binding to glutathione beads at 4 ^°^C for 3 h. The beads were centrifuged at 2500 rpm for 2 min at 4 ^°^C. The beads were washed till the supernatant stops giving color using Bradford reagent. A control with GST only, bound to glutathione beads, was expressed and purified in similar way.

### GST pull-down assay

GST alone and GST-AKT bound to glutathione beads were expressed and purified as described previously. HEK-293T cells were transfected with 2 μg of Myc-Vif for 36 h. The cells were lysed in radioimmunoprecipitation assay buffer. About 10 μg of GST-AKT was incubated with the cell lysate for 4 h at 4 °C. After incubation, the supernatant was discarded, and the beads were washed five to six times with chilled 1× PBS. The beads were boiled in Laemmli buffer and subjected to SDS-PAGE followed by immunoblotting with anti-Myc and anti-GST antibodies.

### *In vitro* AKT kinase assay

HA-AKT and its substrate proteins were transfected in HEK-293T cells. These proteins were purified by immunoprecipitation. Then purified AKT (0.5 μg) was added to the purified substrate protein (2 μg) along with AKT kinase reaction buffer (50 mM Hepes, 0.01% Tween-20, 10 mM MnCl_2_, 1 mM EGTA, 2.5 mM DTT, and 0.1 mM ATP, pH 7.4) for 60 min at 30 °C. All reactions were stopped by adding 5× SDS loading buffer and boiled for 10 min at 95 °C for Western blotting analysis.

### HIV-1 infectivity assay

HEK-293T cells expressing APOBEC3G were cotransfected either with KD-AKT and pNL4-3 or pNL4-3 alone and then treated with AKTi. Treatment with AKTi continued for 72 h post-transfection. The supernatant containing virus was collected after 72 h and used to infect TZMbl cells. The viral infectivity was measured 24 h postinfection in TZMbl by detecting the luciferase activity. TZMbl cells contain luciferase gene downstream of HIV-1 LTR promoter. Luciferase assay was performed as described before (18).

### Statistical analysis

All statistical analyses were performed using Prism 7.0 (GraphPad Software, Inc). The mean ± SEM of all biological replicates was used to make graphs. Statistical analysis was calculated by using two-tailed Student’s *t* test or one-way ANOVA test. *p* Value ≤0.05 was considered significant: ns (not significant) > 0.05; ∗*p* < 0.05, ∗∗*p* < 0.01, and ∗∗∗*p* < 0.001. For Western blot, coimmunoprecipitation, unless indicated otherwise, results are representative of at least three independent experiments.

## Data availability

All data supporting the results are included in the main text file.

## Conflict of interest

The authors declare that they have no conflicts of interest with the contents of this article.
